# Gastritis cystica profunda mimicking submucosal tumor

**DOI:** 10.1055/a-2197-9115

**Published:** 2023-11-14

**Authors:** Li Zeng, Linmao Zheng, Bing Hu, Liansong Ye

**Affiliations:** 1 Department of Gastroenterology and Hepatology, West China Hospital, Sichuan University, Chengdu, P. R. China; 2 Department of Pathology, West China Hospital, Sichuan University, Chengdu, P. R. China

A 69-year-old man presented with a submucosal tumor in the anterior wall of the lower body of the stomach ( Fig. 1 ). He reported no obvious discomfort. His medical history was significant for hypertension, which was controlled by oral administration of amlodipine. Physical examination detected no obvious abnormalities. Endoscopic ultrasound showed a solid cystic lesion, which had a clear boundary and originated from the muscularis mucosae ( Fig. 2 ). Enhanced computed tomography confirmed the presence of a low-density lesion in the stomach ( Fig. 3 ). At the patient’s request, we performed endoscopic resection to determine the nature of the lesion ( Video 1 ). During the procedure, a dual knife was used and submucosal injection was not performed. Complete resection of the lesion was achieved ( Fig. 4 ). There was no bleeding or perforation. Pathology showed cystic dilatation of the gastric glands, with submucosal invasion ( Fig. 5 ). Immunohistochemical studies were negative for CD34 and CD117 but positive for smooth muscle action (SMA) and desmin. Therefore, the diagnosis of gastritis cystica profunda (GCP) mimicking submucosal tumor was made.

**Fig. 1:**
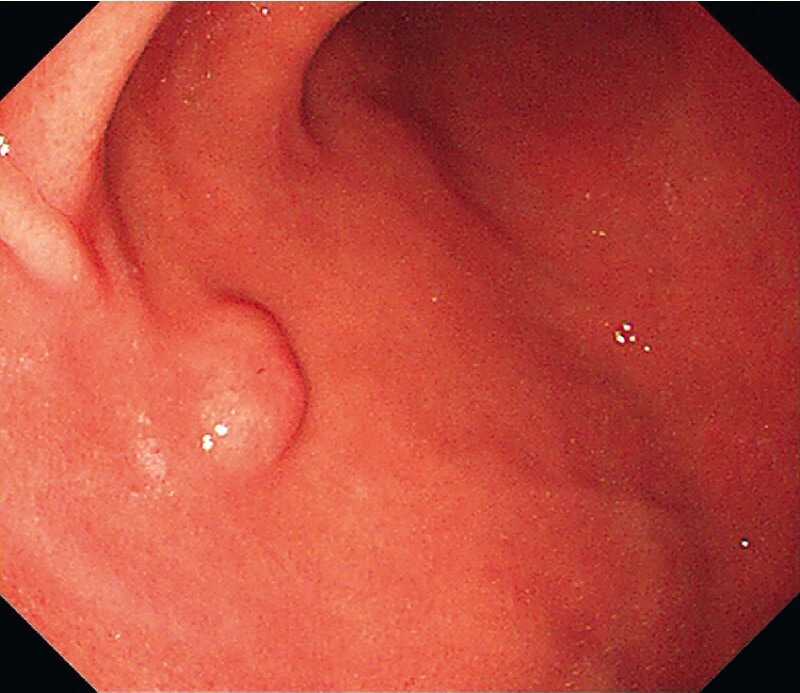
Endoscopy showed a submucosal tumor in the anterior wall of the lower body of the stomach.

**Fig. 2:**
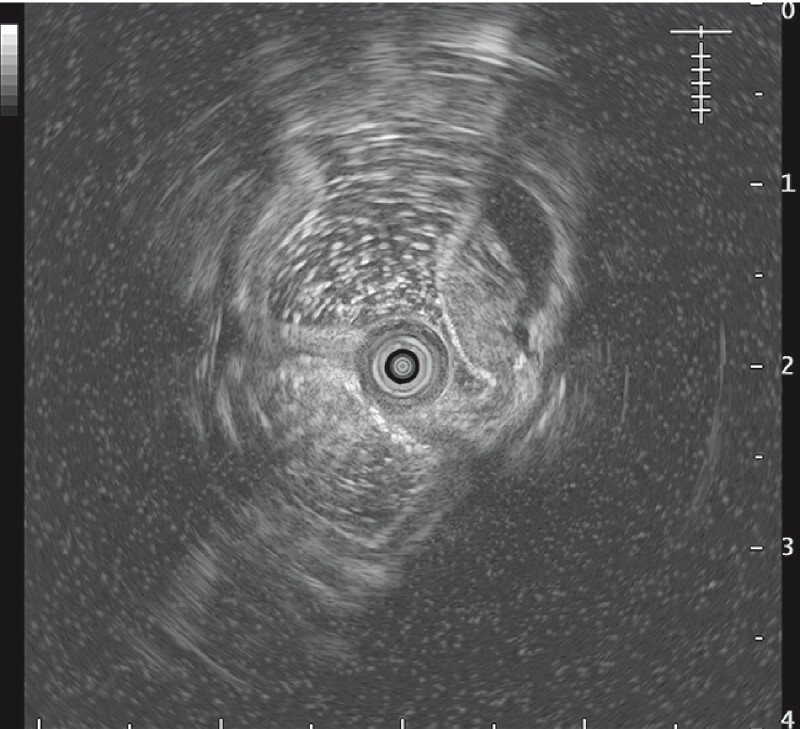
Endoscopic ultrasound confirmed the presence of a solid cystic lesion, which had a clear boundary and originated from the muscularis mucosae.

**Fig. 3:**
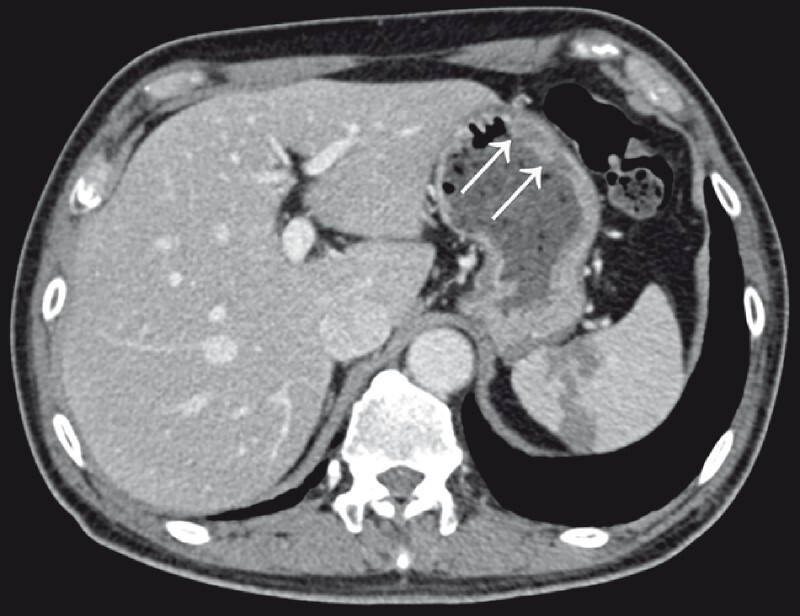
Enhanced computed tomography confirmed the presence of a low-density lesion in the stomach (arrows).

**Video 1** Endoscopic resection of gastritis cystica profunda mimicking a submucosal tumor.


**Fig. 4:**
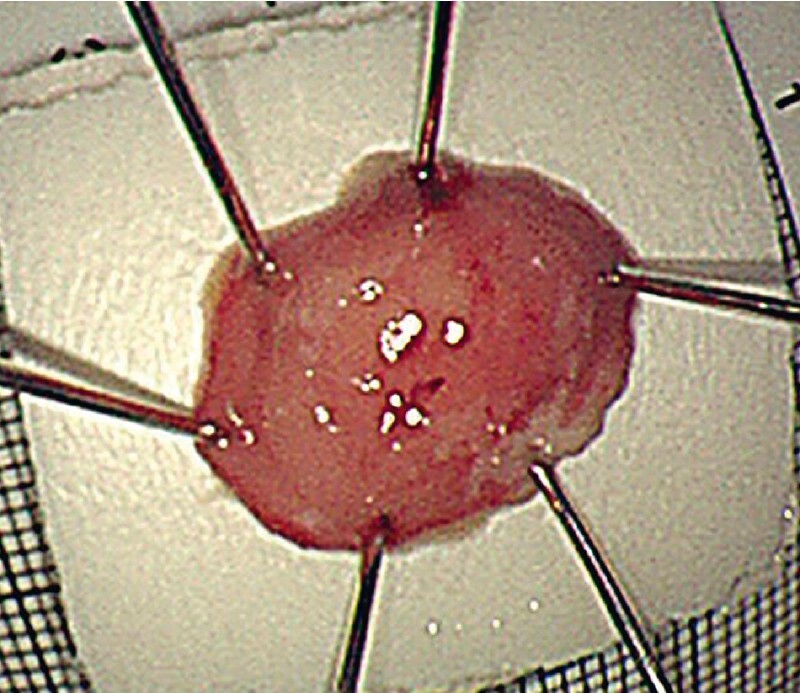
Gastritis cystica profunda: the specimen.

**Fig. 5:**
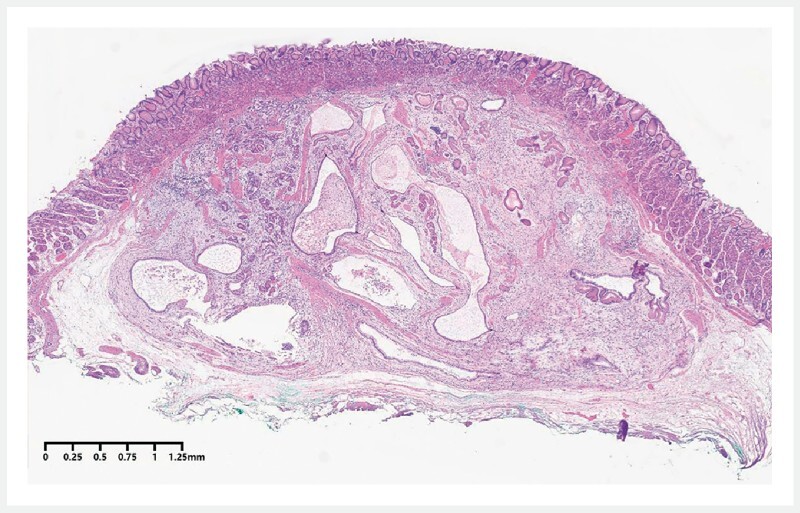
Hematoxylin and eosin staining showed cystic dilation of the gastric glands with submucosal invasion, suggesting gastritis cystica profunda.

The patient fasted for 24 hours and received antibiotic prophylaxis and proton pump inhibitors. He began to drink after 1 day and reported no obvious discomfort. He recovered uneventfully and was discharged after 3 days.

Gastric submucosal tumors are commonly detected during endoscopic examinations. Gastrointestinal stromal tumor and neuroendocrine tumor are common types 1 . In this case, we detected a rare case of GCP mimicking a submucosal tumor, which presented as a solid cystic lesion in the muscularis mucosae and had invaded the submucosal layer. Since GCP may cause abdominal pain and gastrointestinal bleeding, and may be associated with gastric malignant lesions, surgical resection has become an important treatment 2 . Our experience suggests that endoscopic resection can be an alternative for GCP.

Endoscopy_UCTN_Code_CCL_1AB_2AD_3AD

## Notes

Endoscopy E-Videos

https://eref.thieme.de/e-videos
*E-Videos* is an open access online section of the journal *Endoscopy* , reporting on interesting cases and new techniques in gastroenterological endoscopy. All papers include a high-quality video and are published with a Creative Commons CC-BY license. Endoscopy E-Videos qualify for HINARI discounts and waivers and eligibility is automatically checked during the submission process. We grant 100% waivers to articles whose corresponding authors are based in Group A countries and 50% waivers to those who are based in Group B countries as classified by Research4Life (see: https://www.research4life.org/access/eligibility/ ). This section has its own submission website at https://mc.manuscriptcentral.com/e-videos
